# Management of upper intestinal leaks using an endoscopic vacuum-assisted closure system (E-VAC)

**DOI:** 10.1007/s00464-013-3244-5

**Published:** 2013-10-23

**Authors:** M. Bludau, A. H. Hölscher, T. Herbold, J. M. Leers, C. Gutschow, H. Fuchs, W. Schröder

**Affiliations:** Department of General, Visceral and Cancer Surgery, University of Cologne, Kerpener Strasse 62, 50937 Cologne, Germany

**Keywords:** Esophageal perforation, Anastomotic leakage, Endoscopic vacuum-assisted closure system

## Abstract

**Background:**

Esophageal perforations and postoperative leakage of esophagogastrostomy are considered to be life-threatening conditions due to the development of mediastinitis and consecutive sepsis. Vacuum-assisted closure (VAC), a well-established treatment method for superficial infected wounds, is based on a negative pressure applied to the wound via a vacuum-sealed sponge. Endoluminal VAC (E-VAC) therapy is a novel method, and experience with its esophageal application is limited.

**Methods:**

This retrospective study summarizes the experience of a center with a high volume of upper gastrointestinal surgery using E-VAC therapy for patients with leakages of the esophagus. The study investigated 14 patients who had esophageal defects treated with E-VAC. Three patients had a spontaneous defect; two patients had an iatrogenic defect; and nine patients had a postoperative esophageal defect.

**Results:**

The average duration of application was 12.1 days, and an average of 3.9 E-VAC systems were used. For 6 of the 14 patients, E-VAC therapy was combined with the placement of self-expanding metal stents. Complete restoration of the esophageal defect was achieved in 12 (86 %) of the 14 patients. Two patients died due to prolonged sepsis.

**Conclusion:**

This report demonstrates that E-VAC therapy adds an additional treatment option for partial esophageal wall defects. The combination of E-VAC treatment and endoscopic stenting is a successful novel procedure for achieving a high closure rate.

Spontaneous and iatrogenic esophageal perforations as well as postoperative leaks of esophagogastrostomy and esophagojejunostomy are considered to be life-threatening conditions due to the development of mediastinitis and consecutive sepsis [1]. Possible treatment options must drain the septic focus in the mediastinum and close the esophageal wall defect or the dehiscent circular stapler line of the anastomoses.

Self-expanding metal or plastic stents are widely used for these complications and successfully achieve a closure of the defect in most cases [2]. However, in some patients, sepsis persists due to an undrained mediastinal abscess formation, which often is difficult to address by interventional radiologic means. In these cases, surgical resection of the defective esophageal tube or the gastric conduit is required for safe treatment of the mediastinal septic focus and prevention of progressive multiple organ failure [1]. This surgical treatment option severely impairs the patient’s quality of life and usually is associated with a complicated two-stage esophageal reconstruction.

Vacuum-assisted closure (VAC) is a well-established treatment method for superficial infected wounds based on a negative pressure applied to the wound via a vacuum-sealed sponge [3]. The sponge continuously removes wound secretion and interstitial edema, improves microcirculation, and therefore induces an accelerated formation of granulation tissue, resulting in closure of the infected wound.

Clinical experience with application of the VAC system from the endoluminal intestinal side, first attempted for fistulas of rectal anastomoses, has been sparse [4]. This report summarizes the experience of an esophageal high-volume center using endoluminal VAC (E-VAC) for patients with spontaneous, iatrogenic, or postoperative leaks of the tubular esophagus.

## Patients and methods

In this retrospective study, 14 patients (6 women and 8 men) with esophageal defects were treated using the E-VAC between October 2010 and December 2012. The average age of the patients was 67.2 years (range, 43–86 years) (Table 1). The study was approved by the Ethics Committee of the University of Cologne.

**Table 1:** Patients

Type of leak	Etiology	Location	Duration of E-VAC (days)	Number of E-VAC procedure	Endoscopic treatment	Follow-up (days)	Outcome
Iatrogenic perforation	Mediastinoscopy	Middle third	16	5	E-VAC, stent	30	Complete closure
Iatrogenic perforation	Endoscopy, sclerodermia	Middle third	7	2	E-VAC, stent	26	Complete closure
Iatrogenic perforation	Endoscopy	Middle third	15	5	E-VAC	22	Complete closure
Iatrogenic perforation	Thorascopic enucleation of leiomyoma	Distal third	21	6	E-VAC, stent	253	Complete closure
Spontaneous perforation	Boerhaave syndrome	Distal third	21	7	E-VAC	336	Complete closure
Spontaneous perforation	Boerhaave syndrome	Distal third	4	1	E-VAC, stent	–	MOF
Anastomotic leak	Ivor-Lewis esophagectomy	Proximal third	3	1	E-VAC	305	Complete closure
Anastomotic leak	Ivor-Lewis esophagectomy	Proximal third	10	4	E-VAC	12	Complete closure
Anastomotic leak	Ivor-Lewis esophagectomy	Proximal third	23	9	E-VAC, feeding tube	266	Complete closure
Anastomotic leak	Ivor-Lewis esophagectomy	Proximal third	20	5	E-VAC, stent	NA	Complete closure
Anastomotic leak	Ivor-Lewis esophagectomy	Proximal third	13	4	E-VAC	94	Complete closure
Anastomotic leak	Gastrectomy	Distal third	3	1	E-VAC, stent	–	MOF
Anastomotic leak	Gastrectomy	Distal third	7	2	E-VAC, feeding tube	19	Complete closure
Anastomotic leak	Gastrectomy	Distal third	7	3	E-VAC	10	Complete closure

*E-VAC* endoluminal vacuum-assisted closure, *MOF* multiple organ failure, *NA* not available

The primary outcome of the study was leak closure. Complications and side effects of E-VAC therapy also were evaluated (Table 2).

**Table 2:** Overview of published case series

Author	No. of treated patients	Indication	Average no. of E-VAC procedures *n* (range)	Average treatment interval days (range)	Closure rate *n* (%)
Wedemeyer et al. [8]	2	PL	5	15	2/2 (100)
Wedemeyer et al. [9]	8	PL	7	23 (15–31)	7/8 (88)
Ahrens et al. [15]	5	PL	9 (8–12)	28 (24–38)	5/5 (100)
Weidenhagen et al. [11]	6	PL	10 (5–16)	45 (32–84)	5/6 (83)
Loske et al. [13]	14	3 × SP	4 (1–10)	12 (4–31)	13/14 (93)
		3 × IP			
		8 × PL			
Kuehn et al. [16]	9	1 × SP	6 (1–13)	3.5	8/9 (89)
		1 × IP			
		7 × PL			
Schorsch et al. [14]	24	7 × IP	3.7 (1–12)^a^	11 (4–46)	23/24 (96)
		17 × PL			
Brangewitz et al. [17]	32	1 × SP	3.2 (5–28)^a^	23 (9–86)	27/32 (84)
		1 × IP			
		30 × PL			
Bludau et al. [18]	14	3 × SP	3.9 (1–9)	12.1 (3–23)	12/14 (86)
		3 × IP			
		8 × PL			

*E-VAC* endoluminal vacuum-assisted closure, *PL* postoperative leak, *SP* spontaneous perforation, *IP* iatrogenic perforation

^a^Value calculated

The esophageal defects were classified into two groups: iatrogenic and spontaneous perforations (*n* = 6) and anastomotic leakages (*n* = 8). Two patients in the first group had a Boerhaave syndrome, and one patient had a perforation due to a systemic sclerodermia. Iatrogenic perforations occurred during mediastinoscopy (*n* = 1), after thoracoscopic enucleation of a leiomyoma (*n* = 1), and during endoscopic dilation of a benign esophageal stenosis (*n* = 1). Eight patients in the second group experienced an anastomotic leakage after esophagectomy (*n* = 5) or after gastrectomy (*n* = 3).

For 12 patients, the leakage was diagnosed by an endoscopic examination. For two patients, a contrast swallow determined the diagnosis, and a subsequent computed tomography (CT) scan confirmed the existence of the leak.

### E-VAC treatment

All endoscopic interventions were performed either with the patient either under conscious sedation using propofol (Fresenius Kabi, Bad Homburg, Germany) or under general anesthesia.

After the esophageal defect had been located, its size was estimated. In case of a large orifice, the cavity was examined with the endoscope, and an endowasher was used via the working channel of the endoscope to clean the cavity. Then E-VAC therapy was applied by endoscopic insertion of the Endo-SPONGE system (B. Braun Melsungen AG, Melsungen, Germany) through the esophageal defect into the cavity.

**Fig. 1:**
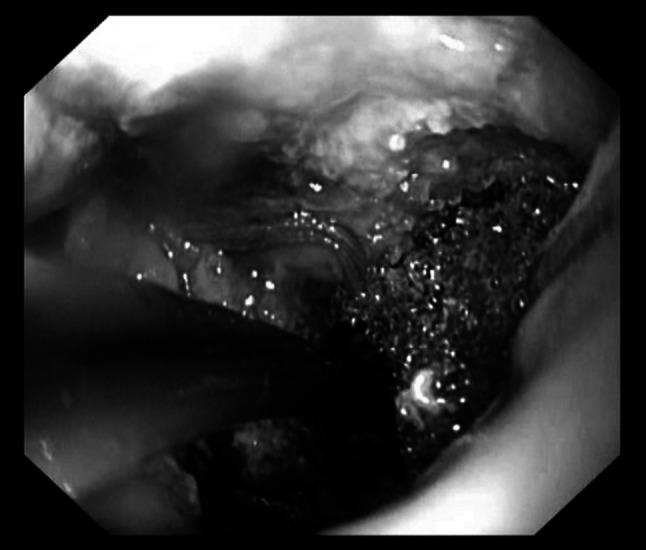
Intracavitary application of endoluminal vacuum-assisted closure (E-VAC)

**Fig. 2:**
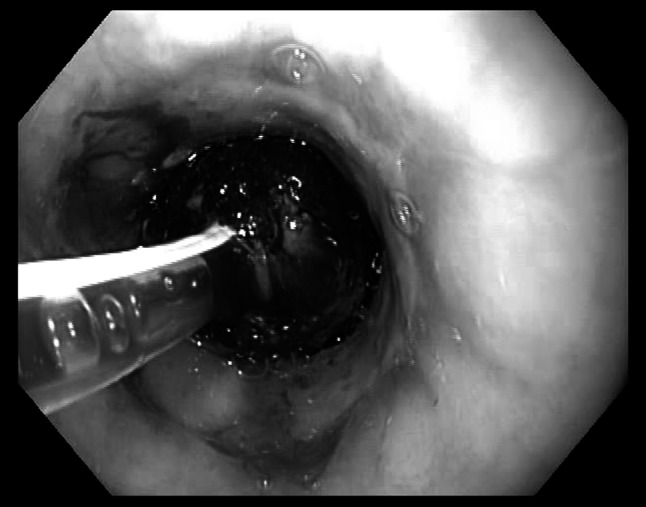
Intraluminal application of endoluminal vacuum-assisted closure (E-VAC)

The Endo-SPONGE consists of an open-pored polyurethane sponge cut to fit into the paraesophageal cavity. The sponge was positioned via an overtube into the region of the leak and placed with the grasper forceps into the paraesophageal cavity (intracavitary vacuum therapy, Fig. 1). In case of a small orifice, the polyurethane sponge was placed at the level of the esophageal wall defect (intraluminal vacuum therapy, Fig. 2). The sponge was connected with a nasogastric tube, and suction was applied to this system by a portable pump. Secretions were continuously evacuated using a negative pressure of 100 mmHg.

After the endoscopic intervention, the patients were awake, spontaneously breathing, and usually capable of being managed on a peripheral ward. The nasogastric tube generally was well tolerated without major problems.

**Fig. 3:**
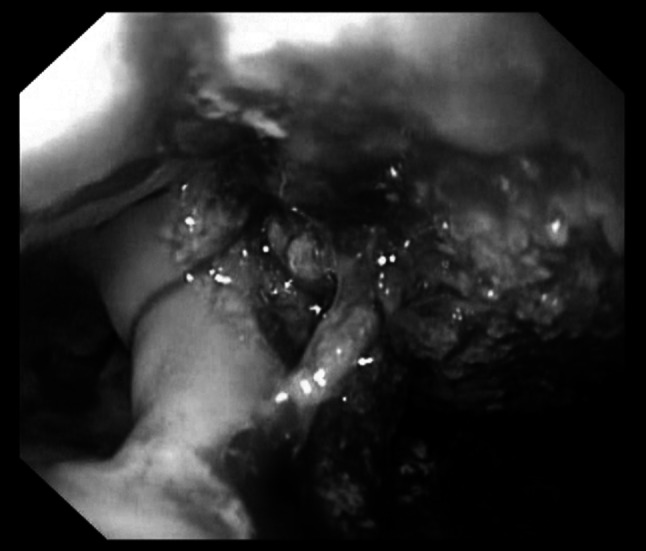
After endoluminal vacuum-assisted closure (E-VAC) treatment

After 2–3 days of continuous suction, the sponge was removed after inactivation of the vacuum by pulling at the nasogastric tube. In case the sponge was adherent to the adjacent tissue, it was removed with aid of an endoscopic forceps. After complete removal, the result of E-VAC therapy was controlled endoscopically (Fig. 3).

In some patients, the procedure was combined with endoscopic stenting of the leakage (Table 1). This was done when E-VAC therapy did not drain any further infectious secretions although the esophageal wall defect was not closed. This persisting leakage was covered with a self-expanding metal stent (SEMS) (Ultrapro 23/28 mm; Boston Scientific, Boston, MA). After a period of 4–6 weeks, the stent was removed.

## Results

Table 1 summarizes the main characteristics of the 14 patients who underwent esophageal E-VAC therapy. The average duration of application was 12.1 days per patient. An average of 3.9 systems per patient were used (interval between changes, 3.1 days; range, 3–5 days). One patient had complete closure of the defect without a change of systems. Complete restoration of the esophageal wall was achieved in 12 (87 %) of the 14 patients.

For 6 of the 14 patients, E-VAC therapy was combined with the placement of SEMS. In all cases, the stent was placed after the E-VAC therapy. The closure was confirmed by a CT scan with oral contrast or a contrast swallow before the patient was allowed to begin oral intake.

For 3 of the 14 patients, enteral feeding was possible with insertion of an endoscopic feeding tube (*n* = 2) or placement of a gastropexy percutaneous endoscopic gastrostomy (PEG) (*n* = 1).

Two patients died due to severe mediastinitis and consecutive sepsis before E-VAC therapy could be successfully completed. The first patient was 87-year old man with Boerhaave syndrome. Due to prolongation of this diagnosis, a septic course developed before drainage of a pleural effusion confirmed the underlying condition. The second patient, a 74-year-old man, had a gastric cancer with gastrectomy and Roux-en-Y reconstruction. The patient experienced anastomotic leakage of the esophagojejunostomy in the lower mediastinum. After confirmation of the diagnosis on postoperative day 5, the patient was transferred to our department. Both patients died due to a therapy-resistant sepsis after prolonged therapy of the esophageal leak.

Follow-up endoscopy was performed for 11 of the 14 patients. The mean follow-up period was 106 days (range, 10–335 days). In two cases, an esophageal stenosis was diagnosed, which was treated successfully by one-time pneumatic dilation. Otherwise, no complications related to the E-VAC therapy were observed.

## Discussion

Esophageal perforations as well as postoperative leaks of esophagogastrostomy and mediastinal esophagojejunostomy usually cause the development of mediastinitis and consecutive sepsis [1, 5]. However, for these esophageal defects, surgical resection was the mainstay for a long period. Interventional mostly endoscopic treatments have replaced surgical techniques.

Endoscopic treatment consists predominantly of applying different endoscopic stents. This treatment has proved to be as effective as surgical resection [2]. In addition, reports describe the instillation of fibrin glue into the leakage until the defect is plugged [6, 7]. This technique is used only for very small leaks.

As a new alternative treatment for esophageal perforation or postoperative leakages, E-VAC therapy was introduced some years ago. The first experience with E-VAC application in the upper gastrointestinal (GI) tract was published as a case report in 2008 [8]. In two patients, the esophageal defect was successfully closed with E-VAC therapy.

In further studies, the technique was modified [9–14] and demonstrated to be a feasible and safe procedure. In 13 (92 %) of 14 patients, Loske et al. [12] showed a successful closure of esophageal leaks with intracavitary or intraluminal placement of the sponge. Only one patient experienced a stenosis during the follow-up period. This complication was confirmed in a small series of five patients with two stenoses that required further endoscopic treatment [15]. In another series, Wedemeyer et al. [9] demonstrated similar success rates with eight patients, and no major complications occurred during a follow-up period longer than 6 months.

Kuehn et al. [16] reported on E-VAC used for nine patients. Four of the nine patients were treated with a hybrid procedure consisting of an initial endoscopic intervention with E-VAC followed by an open revision operation of the thoracic cavity. The mean number of sponge insertions was six, with the sponges changed every 3.5 days. A successful closure was achieved in eight of the nine patients.

Comparison of the published series with our results demonstrated a similar closure rate of 86 %. The average numbers of E-VAC treatments and intervals also were comparable. In our case study, we could prove that patients’ tolerance and comfort were acceptable. The majority of the patients required only sedation for endoscopy and could be managed on a normal ward without intensive care unit (ICU) support. This contrasts with other reports. In the first study of E-VAC therapy, the patients had to be treated in the ICU with mechanical ventilation under general anesthesia [9].

In the latest published study on the treatment of esophageal leaks, the results of 39 patients with a stent (SEMS or self-expanding plastic stent [SEPS]) were compared with 32 patients after E-VAC therapy [17]. The overall closure rate was 84 % in the E-VAC group, which was significant higher than the rate of 54 % in the stent group. However, the characteristics of the patients in the two groups were very different regarding the surgical treatment and anastomotic site. The majority of the patients in the stent group (69 %) underwent esophagogastrostomy, whereas almost 50 % of the patients in the E-VAC group underwent reconstruction via an esophagojejunostomy.

The aforementioned anastomotic types differed completely in terms of vascularization and localization (abdominal vs intrathoracic). In addition, the diagnosed leaks in the E-VAC group were significantly larger, so a meaningful comparison of the two groups is questionable. Taking these drawbacks into consideration, this retrospective analysis mainly demonstrated that a clearcut difference between the two treatment options does not exist currently.

On the other hand, the successful combination of different endoscopic interventions with E-VAC treatment had several advantages. First, enteral feeding was possible with insertion of an endoscopic feeding tube or placement of a percutaneous endoscopic gastrostomy (PEG). The combined use of E-VAC therapy with endoscopic stents was even more important. In all cases, E-VAC was applied to clean the perforation cavity and drain the septic focus. After this, the defect was covered with a stent. With this combined treatment, even large defects could finally be closed.

This study encourages the endoscopic treatment of upper intestinal leaks using different endoscopic techniques. With E-VAC therapy, clinicians have another practical endoscopic tool for draining and cleaning a septic focus of the paraesophageal tissue. It drains septic fluid and enhances tissue healing. In combination with endoscopic stent therapy, E-VAC can shift the border between surgical and endoscopic treatment further toward the less invasive endoscopic intervention.

The limitations of this method are a persisting or even a septic course. This mostly indicates that the septic focus is not adequately drained, and surgical resection should be considered. In these cases, surgical judgment is of uttermost importance so the point of reversible sepsis is not missed.

## Conclusion

This study confirms the feasibility of E-VAC treatment for leakages of the esophageal tube. The use E-VAC extends the spectrum of interventional endoscopy. The combination of E-VAC therapy followed by endoscopic stenting is a novel procedure. This hybrid therapy combines the two surgical treatment strategies for esophageal wall defects: draining the mediastinal or pleural abscess and closing the defect of the esophageal tube.
